# Early embryo achievement through isolated microspore culture in *Citrus clementina* Hort. ex Tan., cvs. ‘Monreal Rosso’ and ‘Nules’

**DOI:** 10.3389/fpls.2015.00413

**Published:** 2015-06-11

**Authors:** Benedetta Chiancone, Marines M. Gniech Karasawa, Valeria Gianguzzi, Ahmed M. Abdelgalel, Ivett Bárány, Pilar S. Testillano, Daniela Torello Marinoni, Roberto Botta, Maria Antonietta Germanà

**Affiliations:** ^1^Dipartimento di Scienze degli Alimenti, Università degli Studi di Parma, ParmaItaly; ^2^Dipartimento di Scienze Agrarie e Forestali, Università degli Studi di Palermo, PalermoItaly; ^3^Centro de Investigaciones Biològicas – Consejo Superior de Investigaciones Científicas, MadridSpain; ^4^Dipartimento di Scienze Agrarie, Forestali e Alimentari, Università degli Studi di TorinoGrugliasco, Italy

**Keywords:** citrus breeding, gametic embryogenesis, homozygosity, isolated microspore culture, meta-Topolin

## Abstract

Microspore embryogenesis is a method of achieving complete homozygosity from plants. It is particularly useful for woody species, like *Citrus*, characterized by long juvenility, a high degree of heterozygosity and often self-incompatibility. Anther culture is currently the method of choice for microspore embryogenesis in many crops. However, isolated microspore culture is a better way to investigate the processes at the cellular, physiological, biochemical, and molecular levels as it avoids the influence of somatic anther tissue. To exploit the potential of this technique, it is important to separate the key factors affecting the process and, among them, culture medium composition and particularly the plant growth regulators and their concentration, as they can greatly enhance regeneration efficiency. To our knowledge, the ability of meta-Topolin, a naturally occurring aromatic cytokinin, to induce gametic embryogenesis in isolated microspores of *Citrus* has never been investigated. In this study, the effect of two concentrations of meta-Topolin instead of benzyladenine or zeatin in the culture medium was investigated in isolated microspore culture of two genotypes of *Citrus*. After 11 months of isolated microspore culture, for both genotypes and for all the four tested media, the microspore reprogramming and their sporophytic development was observed by the presence of multinucleated calli and microspore-derived embryos at different stages. Microsatellite analysis of parental and embryo samples was performed to determine the embryo alleles constitution of early embryos produced in all tested media, confirming their origin from microspores. To our knowledge, this is the first successful report of *Citrus* microspore embryogenesis with isolated microspore culture in *Citrus*, and in particular in *Citrus clementina* Hort. ex Tan, cvs. ‘Monreal Rosso’ and ‘Nules.’

## Introduction

Biotechnology methods can be used to enhance the efficiency of traditional breeding programs. Gametic embryogenesis is a biotechnological tool employed in both basic and applied research. Immature gametes, opportunely induced, can deviate from the normal gametophytic developmental pathway toward the sporophytic one. The sporophytic pathway leads to the production of haploid organisms (Hs), with the gametic chromosome number (n instead of 2n), or doubled haploids (DHs), haploids that underwent, spontaneously or induced, chromosome duplication, becoming homozygous at all *loci*. Gametic embryogenesis techniques and particularly microspore embryogenesis, are efficient methods for obtaining homozygous individuals. They can be used for important breeding applications such as mutation, selection, genetic analysis, transformation, and gene sequencing (Germanà et al., 2013).

Developing homozygous lines is very important in crop improvement programs, particularly for woody plants characterized by long reproductive cycles, a high degree of heterozygosity, large size, and, sometimes, by self-incompatibility (Germanà, 2006, 2009, 2011a,b; Seguì-Simarro, 2010). Woody plants are considered recalcitrant species. Few studies reported successful and efficient microspore embryogenesis protocols for woody species (Höfer, 2004; Ramírez et al., 2004; Barany et al., 2005; Bueno et al., 2005, 2006; Germanà, 2006, 2007, 2009, 2011a; Chiancone et al., 2013; Rodríguez-Sanz et al., 2014; Blasco et al., 2015).

Among the woody recalcitrant fruit producing species, *Citrus*, ranks first worldwide, with 126 million tons of fruit produced during 2013 (FAOSTAT Database, 2014). Clementine is believed to be a ‘Mediterranean’ mandarin × sweet orange hybrid. Particularly, the group of Clementine cultivars is the most representative of the Spanish Citrus industry because of their quality and acceptance by the consumers. Especially, the cv. ‘Nules’ is one of the most cultivated clementine and ‘Monreal Rosso’ (MAR) was obtained by gamma rays mutation at the Research Center for the Citrus and the Mediterranean crops (CRA-ACM, Acireale, CT, Italy). Due to its economical importance, clementine is of great interest to breeders.

Among the *Citrus* microspore embryogenesis reports to date (Germanà et al., 1994, 2005; Germanà, 1997, 2007; Germanà and Reforgiato Recupero, 1997; Germanà and Chiancone, 2003), only one examined isolated microspore culture in several *Citrus* species (lemon, orange, clementine, sour orange, grapefruit) and a related genus (*Poncirus*; Germanà et al., 1996).

Since the first studies of Nitsch (1974) on *in vitro* isolated microspore cultures of *Nicotiana*, considerable research has been done on developing protocols for different species for increasing the frequency of embryogenesis *via* isolated microspore culture (Dunwell, 2010; Prem et al., 2012). Although anther culture is often the method of choice for DH production in many crops, because of its higher efficiency and simplicity, the isolated microspore culture technique provides a better way to investigate the processes of pollen embryogenesis at the cellular, physiological, biochemical, and molecular levels. However, it requires better equipment and more skill than anther culture (Nitsch, 1977; Reinert and Bajaj, 1977; Germanà, 2011a). Also isolated microspore culture avoids the regeneration from somatic anther tissue (Ferrie and Caswell, 2010; Germanà, 2011a,b).

Numerous endogenous and exogenous factors affect the embryogenic response of immature gametes in culture (Smykal, 2000; Wang et al., 2000). Genotype, physiological status and growth conditions of donor plants, stage of gamete development, pre-treatment of the flower buds, culture media and conditions of incubation, and their interactions, are all factors that greatly affect the cell response to the *in vitro* culture (Germanà, 2011a,b).

There is no single standard condition or protocol for obtaining plant formation by isolated microspore culture. Microspores of different species and cultivars within a species can have much different requirements for embryogenic development. For these reasons studies of increasing microspore embryogenesis efficiency, focused on detecting the influence of growth regulators on anther culture and isolated microspore culture in *Citrus* spp. and other fruit crops (Germanà et al., 1996, 2006, 2011; Höfer et al., 1999; Germanà and Chiancone, 2003; Höfer, 2004; Bueno et al., 2005, 2006; Chiancone et al., 2006; Padoan et al., 2011).

Meta-Topolin (mT), a naturally occurring aromatic cytokinin, considered an alternative to benzyladenine (BA), zeatin (ZEA), and kinetin (KIN) in plant tissue culture (Aremu et al., 2012), has been used to increase *in vitro* plant propagation efficiency of several species including *Citrus* (Niedz and Evens, 2011). To our knowledge, this alternative cytokinin has not been used to induce microspore embryogenesis by anther or isolated microspore cultures. Esteves et al. (2014) recently tested meta-Topolin in the regeneration medium of isolated microspore culture of recalcitrant barley genotypes. It increased embryo differentiation into green plants by 2.9-fold.

This study investigated the effect of meta-Topolin as a substitute for benzyladenine or zeatin in the culture media used for generating embryos of *Citrus clementina* Hort. ex Tan., cultivars ‘Monreal Rosso’ and ‘Nules’ when using gametic embryogenesis via isolated microspore culture method.

## Materials and Methods

### Plant Material and Pollen Developmental Stage

Flower buds were harvested in April from trees of *C. clementina* Hort. ex Tan., cvs. ‘Monreal Rosso’ (MAR) and ‘Nules’, grown in a collection orchard (Campo d’Orlèans, Palermo 38°N) of the Università degli Studi di Palermo, Italy. Microspore developmental stage was determined in one anther per flower bud size by 4^′^, 6-diamidino-2-phenylindole (DAPI) staining. Anthers from buds of different sizes were squashed in a few drops of DAPI solution (1 mg/mL) and observed under a fluorescent microscope (Zeiss, Axiophot, Germany). For further experiments, only flower buds of the size containing anthers bearing microspores at the uninucleated/vacuolated stage (3.5–4.0 mm), were selected for culture.

### Flower Bud Sterilization, Microspore Isolation, and Culture

As pre-treatment, flower buds were placed in darkness at 4°C for 1 week. Around 80 flower buds were surface sterilized by immersion for 3 min in 70% (v/v) ethyl alcohol, followed by immersion for 20 min in 25% (v/v) commercial bleach (about 0.5% active chlorine in water) and then rinsed three times with sterile distilled water. Anthers were carefully separated from stamens and put in sterile 0.4 M mannitol solution until the isolation protocol, which was performed following the procedure reported by Kumlehn et al. (2006), with little modifications. Particularly, anthers were used as explants, instead of the entire flowers and the density gradient step was skipped. Isolated microspores were cultured at the concentration of 100,000 microspores per mL. A volume of 1.0 mL was placed into each 3001-type Petri dishes (35 mm × 10 mm, BD Biosciences).

All Petri dishes were put at 26 ± 1°C in the dark for the first 30 days, and then placed under cool white fluorescent lamp (Philips TLM 30W/84, France), with a photosynthetic photon flux density of 35 μmol m^-1^ s^-1^ and a photoperiod of 16 light hours.

### Media Composition

For the culture, the medium (referred as medium P) previously employed in experiments on *Citrus* microspore embryogenesis through isolated microspore culture was used (Germanà et al., 1996; **Table 1**). In this medium, among the other plant growth regulators, several cytokinins are present, particularly BA, ZEA, KIN. To study the effect of mT, it was added in substitution of BA or ZEA at the same concentration (respectively, media: PmT/BA, PmT/ZEA) or at a concentration 10 times higher (respectively, media: PmT/BA10, PmT/ZEA10).

**Table 1:** Media used for ‘Monreal Rosso’ and ‘Nules’ isolated microspore culture.

Components	Media				
	P	PMT/BA	PMT/ZEA	PMT/BA10	PMT/ZEA10
	
	Per liter				
N6 Chu Salts	1X	1X	1X	1X	1X
N&N vitamins	1X	1X	1X	1X	1X
Galactose	18 g	18 g	18 g	18 g	18 g
Lactose	36 g	36 g	36 g	36 g	36 g
Ascorbic acid	500 mg	500 mg	500 mg	500 mg	500 mg
Myoinositol	5 g	5 g	5 g	5 g	5 g
Biotin	500 mg	500 mg	500 mg	500 mg	500 mg
Thiamine	5 mg	5 mg	5 mg	5 mg	5 mg
Pyridoxine	5 mg	5 mg	5 mg	5 mg	5 mg
Coconut water	100 mL	100 mL	100 mL	100 mL	100 mL
Casein	500 mg	500 mg	500 mg	500 mg	500 mg
Serine	100 mg	100 mg	100 mg	100 mg	100 mg
Glycine	2 mg	2 mg	2 mg	2 mg	2 mg
Glutamine	800 mg	800 mg	800 mg	800 mg	800 mg
Malt extract	500 mg	500 mg	500 mg	500 mg	500 mg
2,4-D	0.5 mg	0.5 mg	0.5 mg	0.5 mg	0.5 mg
GA_3_	0.5 mg	0.5 mg	0.5 mg	0.5 mg	0.5 mg
Kinetin	0.5 mg	0.5 mg	0.5 mg	0.5 mg	0.5 mg
Zeatin	0.5 mg	0.5 mg	–	0.5 mg	–
Thidiazuron	0.5 mg	0.5 mg	0.5 mg	0.5 mg	0.5 mg
Benzyladenine	0.5 mg	–	0.5 mg	–	0.5 mg
Meta-Topolin	–	0.5 mg	0.5 mg	5.4 mg	5.6 mg

pH	5.8	5.8	5.8	5.8	5.8

*P, Germanà et al. (1996); N6 Chu salts, Chu (1978); N&N vitamins, Nitsch and Nitsch (1969).*

In particular, for the experiments the following media were tested:

(1) PC (control medium): 0.5 mg/L of BA and 0.5 mg/L of ZEA; (Germanà et al., 1996);
(2) PmT/BA: PC medium without BA + 0.5 mg/L mT;
(3) PmT/ZEA: PC medium without ZEA + 0.5 mg/L mT;
(4) PmT/BA10: PC medium without BA + 5.4 mg/L mT;
(5) PmT/ZEA10: PC medium without ZEA + 5.6 mg/L mT.

Seven replicates for each medium were used, thirty five Petri dishes per cultivar.

Early embryos obtained were transferred onto different solid media (**Table 2**) in the attempt to obtain their germination.

**Table 2:** Solid media tested for embryo germination.

Components	Media				
	E	EE	E/ZEA	E/TDZ	MS/TDZ

	Per liter				
MS salts	1X	1X	1X	1X	1X
MS vitamins	1X	1X	1X	1X	1X
Sucrose	30 g	30 g	30 g	30 g	30 g
Ascorbic acid	500 mg	500 mg	500 mg	500 mg	–
Malt extract	500 mg	500 mg	500 mg	500 mg	–
GA_3_	1 mg	2 mg	1 mg	1 mg	–
Zeatin	–	–	1 mg	–	–
NAA	0.02 mg	0.02 mg	0.02 mg	0.02 mg	–
Thidiazuron	–	–	–	1.0 mg	1.0 mg
Agar	8.5 g	8.5 g	8.5 g	8.5 g	8.5 g

pH	5.8	5.8	5.8	5.8	5.8

*MS, Murashige and Skoog (1962); GA_3_, gibberellic acid; NAA, α-naphthaleneacetic acid.*

### Evaluation of the Microspore Response *In Vitro*, Data Processing, and Statistical Analysis

Petri dishes containing isolated microspores in cultures were weekly observed by an inverted microscope (Zeiss) and a binocular microscope (Leica). Samples of isolated microspores were stained with DAPI and observed by a fluorescence microscope (Zeiss, Axiophot, Germany) to monitor their *in vitro* development, once a month, every month, during the culture. After 7 months of culture, per each medium, 400 microspores DAPI-stained (four replicates with around 100 microspores each) were observed, by a fluorescence microscope (Zeiss, Axiophot, Germany). Different structural features have been observed and registered: microspores uninucleated, binucleated with two equal-size nuclei that had just started the sporophytic pathway, trinucleated, tetranucleated, and multinucleated. Moreover, after 11 months of *in vitro* culture, the number of calli and embryos produced per each Petri dish was registered, using a binocular microscope. These values were used to calculate means. Statistical analysis was carried out using SYSTAT 13 software. Two factors were considered: “Cultivar” and “Culture medium,” and differences between them were tested by two-way analysis of variance (ANOVA), at *p* ≤ 0.05 level. Tukey’s test was, then, used to separate means.

### Fixation and Processing for Microscopic Analysis

*In vitro* cultures containing microspores and microspore-derived structures were fixed in 4% paraformaldehyde in phosphate buffered saline (PBS), overnight, at 4°C. After fixation, microspore culture samples were embedded in gelatin, washed in PBS, dehydrated through an acetone series, infiltrated and embedded in Technovit 8100 acrylic resin (Kulzer, Germany), at 4°C, as previously described by Solís et al. (2008). Staining solution of 0.075% toluidine blue in water, was applied on Technovit semithin sections (1 μm) for 10–15 min. After rinsing and drying, preparations were mounted in Eukitt and observed under bright field for structural analysis in a light microscope Zeiss 68105 equipped with a Leica Microsystems DFC420C digital camera.

### Allelic Pattern Detection by SSR Analysis

The allelic pattern was checked on the embryos obtained from *C. clementina* cultivar ‘Monreal Rosso’ and ‘Nules’ isolated microspore culture. DNA was extracted from leaves of the mother plant and from the embryos obtained by *in vitro* culture and collected from the medium by an insulin needle. The samples were frozen in liquid nitrogen and ground using steel beads and a Tissuelyser (QIAGEN^®^, Germany). DNA extraction was performed as described by Doyle and Doyle (1987). The parent DNA was resuspended in 60 μL TE buffer (Tris-EDTA, pH 8.00) and then diluted to 10 ng/μL. Embryo DNA was resuspended in 25 μL TE.

Ten microsatellite loci isolated by Novelli et al. (2006) from *C. sinensis* and by Froelicher et al. (2008) from *C. reticulata* were preliminarly screened on the DNA from the leaves and one was selected for its heterozygosity in the parental genotype: CCSM147 (Novelli et al., 2006). This locus was used for assessing the allelic pattern of the embryos.

Polymerase chain reactions (PCRs) were performed in two steps in a total volume of 10 μl containing 3 μL DNA (corresponding to 30 ng of DNA for the parent plants), 0.25 U of KAPA Taq DNA polymerase (KAPABIOSYSTEMS, Wilmington, MA, USA) 1 μL of 10X PCR buffer, 200 μM nucleotide mix and 0.5 μM of each primer. PCR conditions were as follows: an initial denaturation step at 95°C for 3 min followed by 34 cycles of denaturation (30 s at 95°C), annealing (45 s at 55°C), and extension (90 s at 72°C). The final elongation step was at 72°C for 30 min. Four μL of the product from the first amplification were then used as template for a second PCR, carried out for 28 cycles with the same conditions of the first one.

Polymerase chain reaction products were then analyzed on a 3130 Genetic Analyzer (Applied Biosystems, Foster City, CA, USA). Data were processed using GeneMapper Software (ver. 4.0; Applied Biosystems) and alleles were defined by their size in base pairs, by comparison with the standard size (GeneScan-500 LIZ, Applied Biosystems).

## Results

**FIGURE 1:**
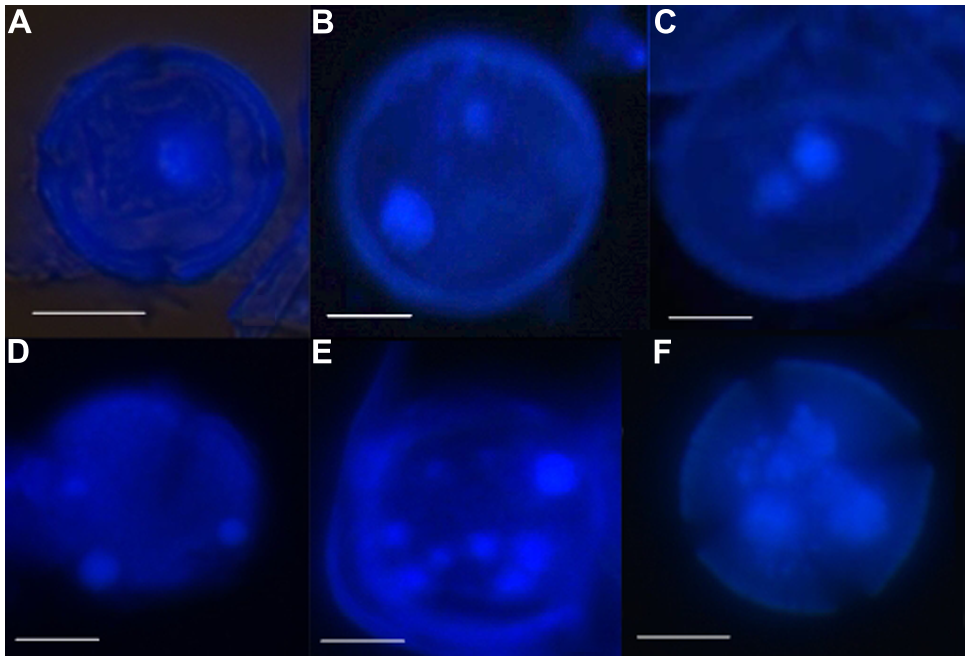
**Nuclei divisions and formation of microspore-derived multicellular structures during early microspore embryogenesis through isolated microspore culture of *Citrus clementina* Hort. ex Tan, cv. ‘Monreal Rosso’ and ‘Nules’, monitored by DAPI staining.(A)** Uninucleated microspore of ‘Monreal rosso’; **(B)** binucleated pollen, originated by asymmetrical division of Nules; **(C)** binucleated microspore, originated by symmetrical division, Nules; **(D)** trinucleated microspore of Monreal Rosso; **(E,F)** multicellular microspore of ‘Monreal Rosso’ **(E)** and ‘Nules’ **(F)**. Bars represent 10 μm.

Using the above methods allowed facilitated observation of the entire microspore embryogenesis process in clementine isolated microspore culture. Monitoring of the culture samples by DAPI staining (to show the nuclei) revealed that initially microspores of both genotypes were mainly uninucleated/vacuolated (**Figure 1A**) This is the developmental stage reported as being the most responsive for embryogenesis induction in clementine (Ramírez et al., 2003) and many other woody and herbaceous species (Germanà and Chiancone, 2003; Germanà et al., 2011; Solís et al., 2008; Prem et al., 2012). It was possible to observe that some microspores did not show any change in the nuclei number or shape. In other microspores the nucleus started to divide. This rarely occurred asymmetrically, i.e., following the normal gametophytic pathway (**Figure 1B**). In most binucleate microspores, the two nuclei are similar in size and shape (**Figure 1C**), indicating their origin by a symmetric division. This type of division is considered the first step of the sporophytic pathway followed by the reprogrammed microspores in microspore embryogenesis (Germanà, 2011a,b). Many microspores followed this pathway and underwent subsequent divisions, so that, later trinucleated (**Figure 1D**), tetranucleated and multinucleated microspores (**Figures 1E,F**) were detected in DAPI-stained squash preparations.

**FIGURE 2:**
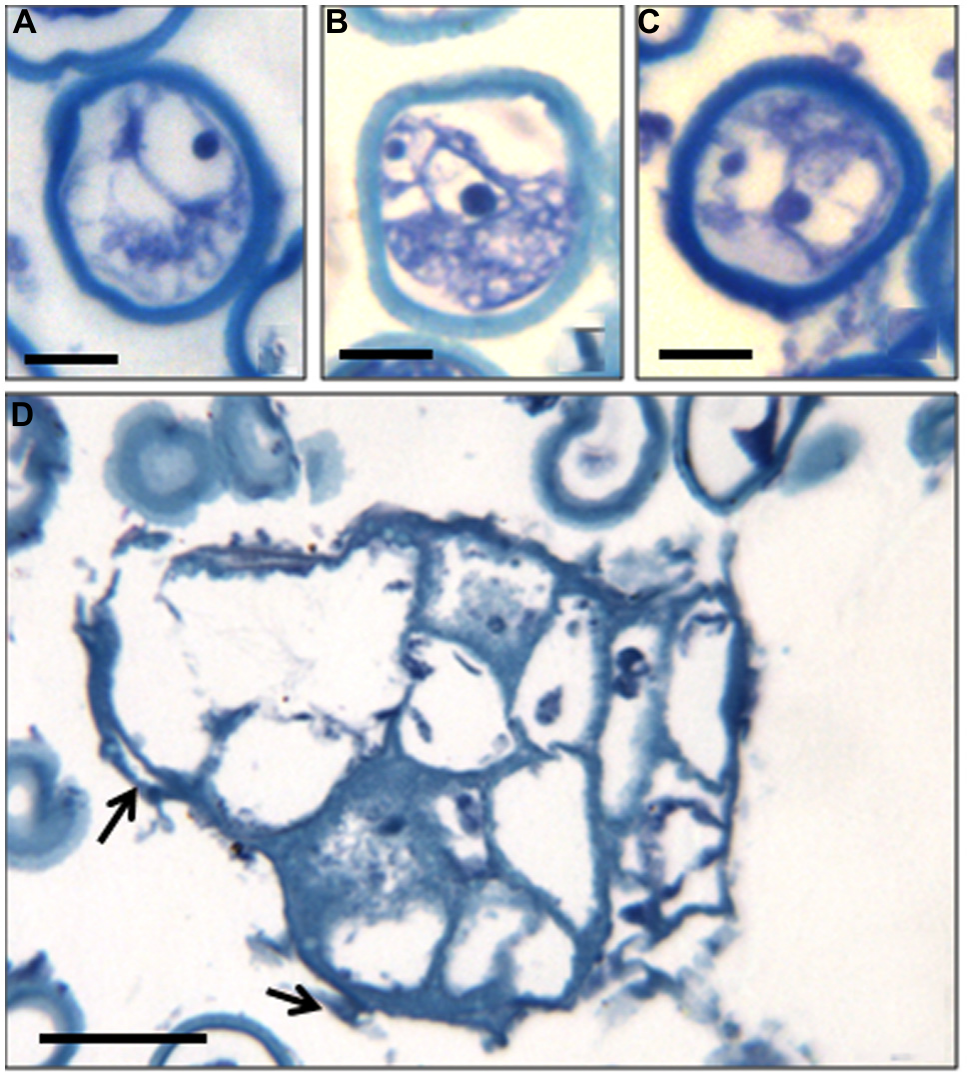
**Cellular structural organization at early microspore embryogenesis through isolated microspore culture of *C. clementina* Hort. ex Tan, cv. ‘Monreal Rosso’ and ‘Nules.’** Toluidine blue-staining of resin sections observed under bright field microscopy. **(A)** Vacuolated microspore at the beginning of the culture, ‘Monreal Rosso’; **(B,C)** Two-celled microspores, ‘Nules’; **(D)** Microspore-derived multicellular structure (in the center) and some dead microspores (at the top), ‘Monreal Rosso.’ Bars represent, in **(A–C)**: 10 μm, in **(D)**: 50 μm.

The structural organization of these microspores and multinuclear structures observed in the cultures were analyzed on semithin sections (**Figure 2**). Samples of the *in vitro* culture were fixed and processed for further microscopical analysis. At culture initiation, the microspores exhibited the typical architecture of the vacuolated microspores, with one nucleus located at the periphery and a central vacuole (**Figure 2A**). At later stages, in toluidine blue-stained sections, developing microspores exhibited differential features, some of them showing two nuclei with similar size and organization, and dense cytoplasms (**Figures 2B,C**), in contrast with the two different nuclei of the bicellular pollen developed *in vivo*. These two-celled structures indicated that the microspores *in vitro* underwent a symmetrical division and switched from their gametophytic developmental pathway toward proliferation; the result of the first embryogenic division of the microspore still exhibiting the exine wall (**Figures 2B,C**). Some dead (empty) microspores with irregular shapes were also observed in the cultures, together with larger multicellular structures (**Figure 2D**). They were elongated structures formed by more or less polygonal cells showing one nucleus and low-dense cytoplasm and vacuoles, and separated by straight cell walls (**Figure 2D**). At the periphery of some of these multicellular structures, remnants of the exine, could be found (arrows in **Figure 2D**). These multicellular microspore-derived structures or proembryos resembled those found in other woody and herbaceous species. The evolution of the *in vitro* system described here, from two-cell and multicellular microspores to large multicellular structures or proembryos indicated that the reprogramming of the microspore and the first steps of the embryogenic pathway were achieved.

Results recorded after 7 months of microspore culture, and their statistical analysis are reported in **Table 3**. No statistically significant differences were detected among treatments of the percentages of uninucleated and binucleated microspores. Moreover, for both cultivars, the percentage of uninucleated microspores with no division was rather high (41.2% for MAR and 46.7% for ‘Nules’). For the trinucleated microspores, a significant interaction was recorded between the two factors, “Cultivar” and “Culture medium,” in which the main factor inducing variability was “Cultivar.” Actually, the medium in which mT replaced ZEA at the same concentration, induced the highest response in MAR (19.1%) and the worst in ‘Nules’ (5.6%; data not shown).

**Table 3:** Influence of cultivar and medium composition on two clementine cultivars, ‘Monreal Rosso’ and ‘Nules’, isolated microspore development, after 7 months (uninucleated, binucleated, trinucleated, multinucleated microspores) and 11 months (calli and embryos) of culture.

Factors		Uninucleated microspores (%)	Binucleated microspores (%)	Trinucleated microspores (%)	Multinucleated microspores (%)	Calli/Petri dish* (n°)	Embryos/Petri dish (n°)
Cultivar	Monreal Rosso	41.2 a	30.7 a	14.2 a	13.9 a	3.7 a	1.0
	Nules	46.7 a	32.3 a	9.7 b	11.2 a	2.9 a	1.5
Cultivar		0.088	0.359	0.002	0.548	0.090	
Medium	PC	41.9 a	30.8 a	11.5 a	15.9 a	4.0 ab	1.2
	PmT/BA	43.1 a	27.3 a	13.2 a	16.4 a	4.4 a	1.4
	PmT/ZEA	41.4 a	32.8 a	12.4 a	13.5 ab	2.5 ab	1.0
	PmT/BA10	44.7 a	34.2 a	13.0 a	8.2 b	2.4 b	1.4
	PmT/ZEA10	48.6 a	32.6 a	9.7 a	9.1 ab	3.2 ab	1.4
Culture medium		0.615	0.153	0.465	0.007	0.019	–
Cultivar × Culture medium		0.208	0.381	0.024	0.383	0.254	–

Two-way ANOVA, Tukey’s test, *p* ≤ 0.05.

*Average number of calli recorded per each medium and per each cultivar (seven Petri dishes/medium/cultivar).

*PC (control medium), 0.5 mg/L BA and 0.5 mg/L ZEA (Germanà et al., 1996); PmT/BA, PC medium without BA + 0.5 mg/L mT; PmT/ZEA, PC medium without ZEA + 0.5 mg/L mT; PmT/BA10, PC medium without BA + 5.4 mg/L mT; PmT/ZEA10, PC medium without ZEA + 5.6 mg/L mT.*

*Per each factor and per each column, values followed by different letters are statistically different.*

The primary factor influencing induction of multinucleated microspores was “Culture medium.” Tukey’s test evidenced that the control medium (PC) and PmT/BA induced a statistically higher percentage (15.9 and 16.4% respectively) of multinucleated microspores, while the mT/BA10 medium the lowest (8.2%). For the other tested media, the percentages of multinucleated microspores were intermediate between the reported values (**Table 3**).

**FIGURE 3:**
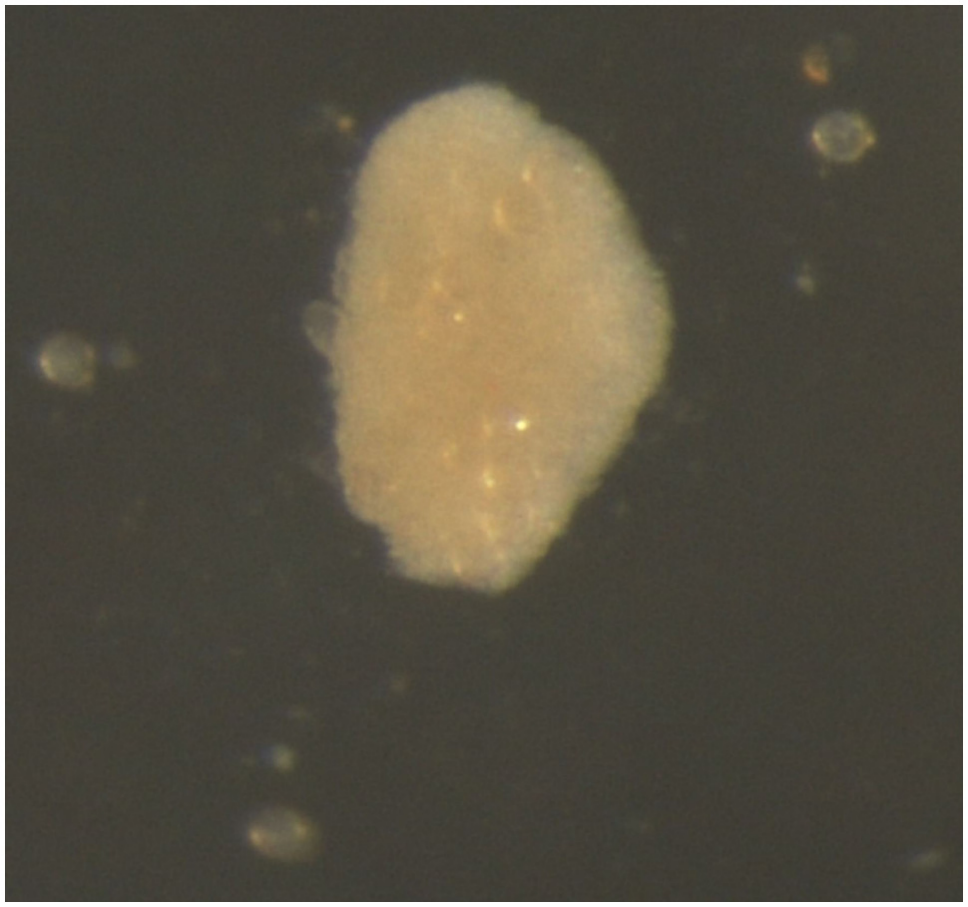
**Microspore-derived callus of ‘Monreal Rosso’ in the PmT/BA medium**.

After 5 months of culture, binocular microscope observations revealed new structures: light brown calli (**Figure 3**) that increased in quantity and volume during the culture. A statistical analysis of number of calli per Petri dish after 11 months of culture, demonstrated that the culture medium was the also primary factor that influenced the microspore response of this parameter. As with multinucleated microspores, the PmT/BA and PmT/BA10 media treatments produced statistically significant differences between the average number of calli/Petri dish (4.4 and 2.4, respectively; **Table 3**).

**FIGURE 4:**
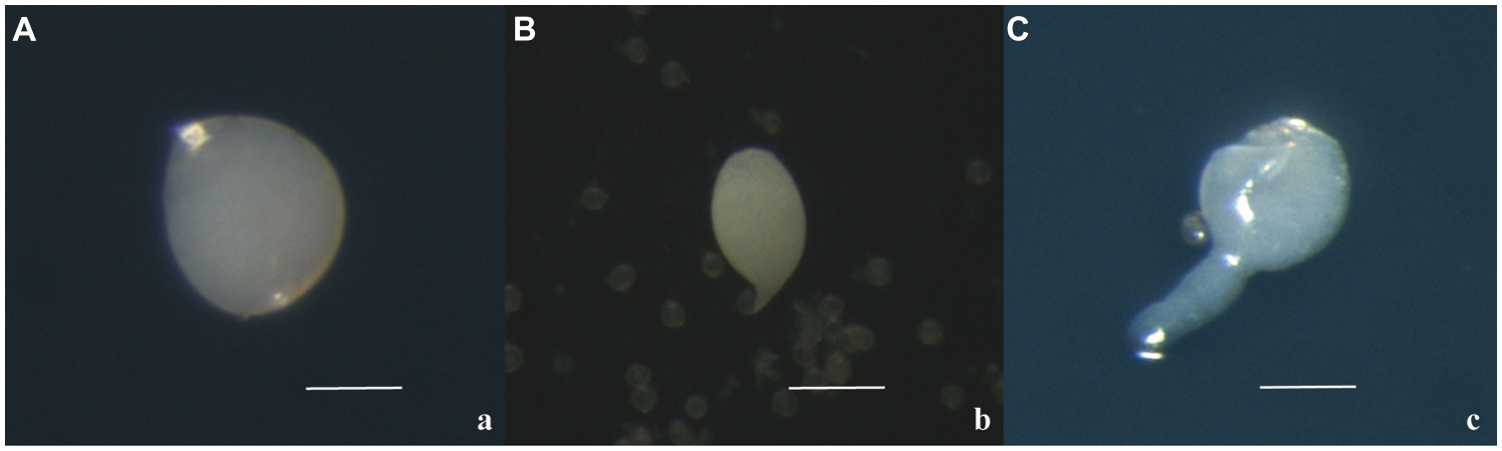
**Microspore-derived embryos of ‘Nules’ **(A)**, ‘Monreal Rosso’ **(B)** and ‘Nules’ **(C).** Bars represent 100 μm**.

Together with calli, the formation of globular embryos was detected: they were pearl white and round (**Figure 4A**). During the culture, the round embryos elongated, often with a suspensor-like structure (**Figures 4B,C**). This kind of structure has not previously observed in the microspore-derived embryos obtained through *Citrus* anther culture.

Embryo production was observed for both cultivars and for all media tested. This is the first report of gamete-derived embryos obtained by isolated microspore culture in *Citrus*. Differences were recorded between the cultivars, with the ‘Nules’ cultivar showing a higher average number of embryos/Petri dish regenerated than in MAR (1.5 vs. 1.0; **Table 3**). However, while the two cultivars responded differently to the five different media, it appears that the higher concentration of mT, replacing BA or ZEA, was not detrimental for the embryo induction. The best responses were induced for MAR in the media PC and PmT/BA10 (1.3) and for ‘Nules’ in the media PmT/BA and PmT/ZEA10 (1.8; data not shown).

**FIGURE 5:**
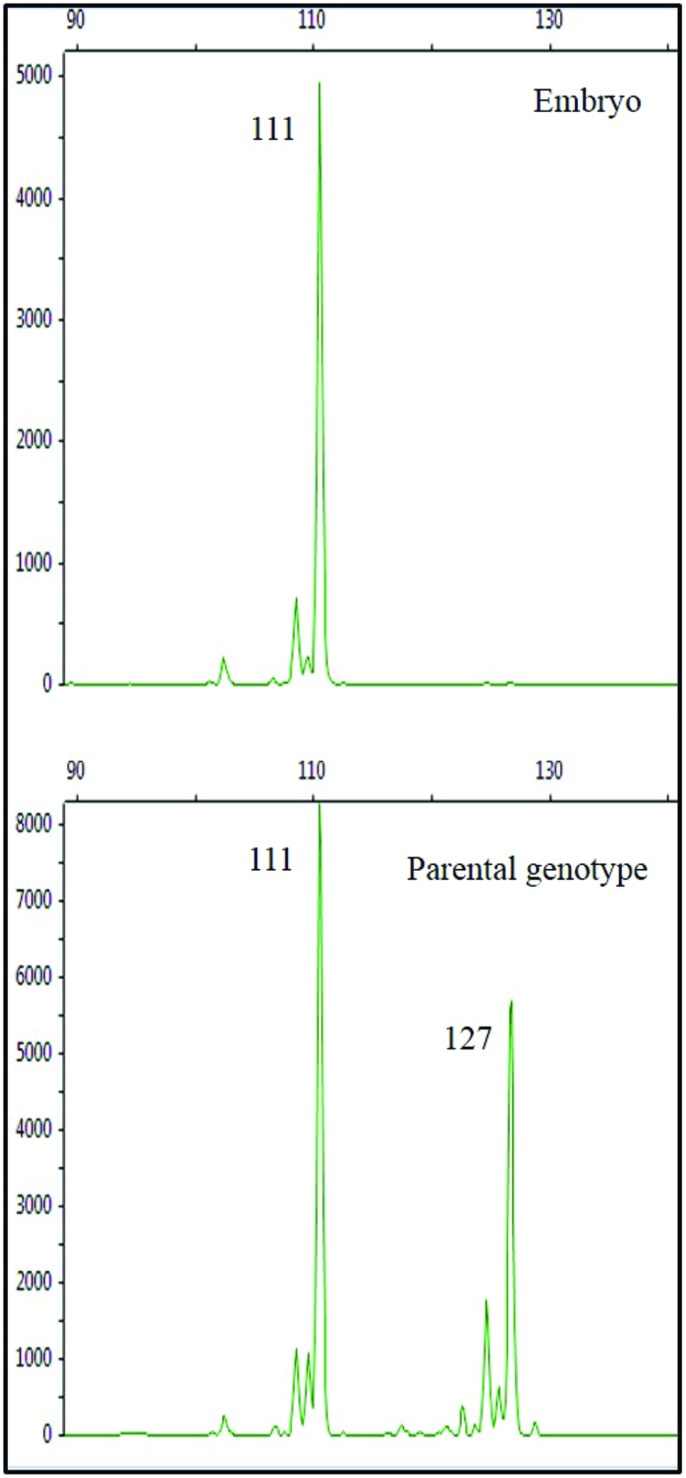
**Amplicons of the SSR locus CCSM147 in the embryo (top) and in the parental genotype (bottom) cv. ‘Monreal Rosso.’ Values in box beside each peak represent the allele size (bp).** The allelic pattern of the embryo shows a single allele, shared with the parental genotype.

The results of the analysis at the SSR locus CCSM 147 showed a clear amplification: while the parental genotype was heterozygous, the allelic pattern of the embryos showed a single allele, shared with the parental genotype (**Figure 5**). This result is a first step in confirming the origin of the embryos from the ‘Monreal Rosso’ and ‘Nules’ gametophyte, although it was not yet possible, due to their small size, to check the ploidy condition of the embryos (either haploid or double haploid).

The embryos obtained were transferred from liquid to different solid media to achieve germination and plantlet production. After 12 months of trials with several media (**Table 2**), no germination was observed in microspore-derived embryos, probably due to dormancy caused by immaturity. Physiological and biochemical aspects of these microspore-derived embryos should be investigated to determine if the lack of endosperm, medium composition or dormancy prevent germination. Further investigations are in progress to obtain embryo conversion testing different factors, such as exposure to cold temperature and/or drying.

## Discussion

The great potential of haploids and gametic embryogenesis in woody plant breeding has been well-demonstrated. Haploids can improve the efficiency and the speed of laborious and time-consuming traditional breeding methods. While *in vitro* isolated microspore culture is a standard breeding method in many crops, such as *Brassicaceae* and cereals, this technique has not been exploited in fruit crops because the induction frequency is low, plant recovery is difficult and response is highly genotype-dependent (Höfer et al., 1999; Bueno et al., 2005, 2006). Earlier work with isolated microspore culture of several *Citrus* species (lemon, orange, clementine, sour orange, and grapefruit) and a related genus (*Poncirus*) have been reported by Germanà et al. (1996). Multi-nucleated structures, pseudobulbils and small proembryos, were obtained but which failed to develop significantly.

In *C. clementina* Hort. ex Tan., the gametic embryogenesis process through isolated microspore culture, is slower than in other herbaceous and woody species, requiring up to 5 months for the first callus tissue or embryos. However, the microspores continued regenerating embryos and calli for 11 months in culture. In *Brassica* isolated microspore culture, first embryos are usually observed within 2 weeks of culturing (Barro and Martin, 1999), and in wheat after 9–12 days (Hu and Kasha, 1999). In fruit tree crops, apple embryo regeneration through isolated microspore culture, was observed after 8–12 weeks (Höfer et al., 1999). In olive, Bueno et al. (2005) reported the first pro-embryos after 4 weeks.

The media supplemented with mT showed microspore switching from the gametophytic to the sporophytic pathway as well as the PC medium. However, it appears that the response to mT in the media, as reported in numerous experiments, is genotype-dependent. For example, ‘Nules’ embryo production seems be favored by mT addition. Possibly mT replaces both BA and ZEA, at the same concentration, giving rise to embryo regeneration rate comparable to that of the control. In ‘Nules’, replacing BA with mT 10-fold more concentrated was not beneficial for the induction of multinucleate microspores and calli. However, for embryo induction, adding a higher concentration of mT as replacing BA did not affect the regeneration rate.

The results here reported indicate that meta-Topolin can be employed not only in *Citrus* micropropagation (Niedz and Evens, 2011), but also in the microspore embryogenesis process. The effect of mT on microspore embryogenesis induction could be due to its anti-senescence activity and plant growth stimulation activity. Other cytokinin-like compounds, such as polyamines (PAs), considered potent senescence inhibitors (Altman et al., 1977; Kaur-Sawhney et al., 1980) and implicated in several plant growth and development processes (Torrigiani et al., 1989; Bagni and Tassoni, 2001), improved the embryogenic callus production through anther culture in *C. clementina* Hort. ex Tan. (Chiancone et al., 2006). To understand how anti-senescence substances influence microspore embryogenesis induction could facilitate understanding the mechanisms beyond this phenomenon as well as being used to increase the efficiency of breeding programs.

Actually, an effective regeneration system through isolated microspore culture could facilitate male gametophytic selection (MGS) in *Citrus*. This strategy would allow early genotype screening for selection of desirable alleles on pollen grains (Clegg et al., 1978; Hormaza and Herrero, 1996; Ravikumar et al., 2007). With respect to the sporophytic selection, the MGS has advantages for selecting among very high numbers of haploid individuals in a small space (Soleimani et al., 2010), allowing selection also of recessive characters and mutations, without the interference of dominance. Furthermore, as pollen is the result of genetic recombination, possibly new allelic combination and mutations can be selected for physiological and biochemical characteristics by applying stress during microspore culture (Evans et al., 1990).

## Conclusion

The characteristics of angiosperm pollen (haploidy, small size, great number, totipotency) make it very useful in biotechnology as immature microspores can be manipulated to improve the efficiency, rapidity, and precision of plant breeding methods. The *in vitro* culture of immature microspores is a good way to recover homozygosity via embryogenesis in higher plants. The potential value of isolated microspore culture in higher plants is obvious. However, a well-defined and efficient procedure of regeneration through microspore embryogenesis is necessary.

The results here presented are a major step in understanding *C. clementina* Hort. ex Tan. microspore embryogenesis. Actually, this is first report of regeneration of microspore-derived embryos through isolated microspore culture of the two clementine cultivars ‘Monreal Rosso’ and ‘Nules.’ Additional investigations are needed to optimize the medium composition and increase the regeneration rate. Studies to promote the development of obtained embryos and recover plantlets from them are now in progress.

## Author Contributions

BC statistically analyzed the data and wrote the first draft of the manuscript. AA, MK, and VG performed the experiments. BC and MK contributed to the design of the work. IB and PT processed the samples, elaborated, and interpreted the results of the microscopic analysis. DM and RB processed the samples, analyzed, and interpreted the results of the molecular marker analyses. MG designed the research and coordinated the project, drafted and revised the manuscript, and is corresponding author. All authors collaborated in the revising of the manuscript. All authors read and approved the manuscript.

## Conflict of Interest Statement

The authors declare that the research was conducted in the absence of any commercial or financial relationships that could be construed as a potential conflict of interest.

## References

[B1] Altman A. Kaur-Sawhney R. Galston A. W. (1977). Stabilization of oat leaf protoplasts through polyamine mediated inhibition of senescence. *Plant Physiol.* 60 570–574. 10.1104/pp.60.4.570 16660139 PMC542665

[B2] Aremu A. O. Bairu M. W. Doležal K. Finnie J. F. Van Staden J. (2012). Topolins: a panacea to plant tissue culture challenges? *Plant Cell Tiss. Org. Cult.* 108 1–16. 10.1007/s11240-011-0007-7

[B3] Bagni N. Tassoni A. (2001). Biosynthesis, oxidation and conjugation of aliphatic polyamines in higher plants. *Amino Acids* 20 301–317. 10.1007/s007260170046 11354606

[B4] Barany I. González-Melendi P. Mityko J. Fadón B. Risueño M. C. Testillano P. S. (2005). Microspore-derived embryogenesis in *Capsicum annuum*: subcellular rearrangements through development. *Biol. Cell* 97 709–722. 10.1042/BC20040142 15910280

[B5] Barro F. Martin A. (1999). Response of different genotypes of *Brassica carinata* to microspore culture. *Plant Breed*. 118 79–81. 10.1046/j.1439-0523.1999.118001079.x

[B6] Blasco M. Badenes M. L. Naval M. (2015). Embryogenic response from anther culture of cultivars of loquat (*Eriobotrya japonica* (Thunb.) Lindl.) from different origins. *Euphytica* 10.1007/s10681-015-1386-3

[B7] Bueno M. A. Pintos B. Hofer M. Martin A. (2005). Pro-embryos induction from *Olea europaea* L. isolated microspore culture. *Acta Physiol. Plant.* 27 695–701. 10.1007/s11738-005-0073-8

[B8] Bueno M. A. Pintos B. Martin A. (2006). Induction of embryogenesis via isolated microspore culture in *Olea europaea* L. *Olivebioteq* 1 9–25.

[B9] Chiancone B. Tassoni A. Bagni N. Germanà M. A. (2006). Effect of polyamines on in vitro anther culture of *Citrus clementina* Hort. ex Tan. *Plant Cell Tiss. Org.* 87 145–153. 10.1007/s11240-006-9149-4

[B10] Chiancone B Testillano P. Risuno M. C. Mohamed A. Padoan D. Khan P. S. S. (2013). Coltura in vitro di microspore isolate per il miglioramento genetico di *Olea europaea* L. *Acta Italus* *Hortus* 10 51–55.

[B11] Chu C. (1978). “The N_6_ medium and its applications to anther culture of cereal crops,” in *Proceedings of Symposium on Plant Tissue Culture*, Beijing, 43–50.

[B12] Clegg M. T. Kahler A. L. Allard R. W. (1978). Estimation of lifecycle components of selection in an experimental plant population. *Genetics* 89 765–792. 17248851 10.1093/genetics/89.4.765 PMC1213867

[B13] Doyle J. J. Doyle J. L. (1987). A rapid DNA isolation procedure for small quantities of fresh leaf tissue. *Phytochem. Bull.* 19 11–15.

[B14] Dunwell J. M. (2010). Haploids in flowering plants: origins and exploitation. *Plant Biotechnol. J.* 8 377–424. 10.1111/j.1467-7652.2009.00498.x 20233334

[B15] Esteves P. Clermont I. Marchand S. Belzile F. (2014). Improving the efficiency of isolated microspore culture in six-row spring barley: II-exploring novel growth regulators to maximize embryogenesis and reduce albinism. *Plant Cell Rep.* 33 871–879. 10.1007/s00299-014-1563-1 24519013

[B16] Evans D. E. Singh M. B. Knox K. B. (1990). “Pollen development: applications in biotechnology,” in *Microspores: evolution and ontogenty* eds Blackmore S. Knox R. B. (London: E. Academic Press), 309–338.

[B17] Ferrie A. M. R. Caswell K. L. (2010). Isolated microspore culture techniques and recent progress for haploid and doubled haploid plant production. *Plant Cell Tiss. Org.* 104 301–309. 10.1007/s11240-010-9800-y

[B18] FAOSTAT Database. (2014). Available at: http://faostat.fao.org/default.aspx

[B19] Froelicher Y. Dambier D. Bassene J. B. Costantino G. Lotfy S. Didout C. (2008). Characterization of microsatellite markers in mandarin orange (*Citrus reticulata* Blanco). *Mol. Ecol. Resour.* 8 119–122. 10.1111/j.1471-8286.2007.01893.x 21585732

[B20] Germanà M. A. (1997). “Haploidy in Citrus,” in *In Vitro Haploid Production in Higher Plants*, eds Jain S. M. Sopory S. K. Veilleux R. E. (Dordrecht: Kluwer Academic Publishers), 5 95–217.

[B21] Germanà M. A. (2006). Doubled haploid production in fruit crops. *Plant Cell Tiss. Org.* 86 131–145. 10.1007/s11240-006-9088-0

[B22] Germanà M. A. (2007). “Haploidy,” in *Citrus: Genetics, Breeding and Biotechnology*, ed.Khan I. (Wallingford: CABI), 167–196. 10.1079/9780851990194.0167

[B23] Germanà M. A. (2009). “Haploid and doubled haploids in fruit trees,” in *Advances in Haploid Production in Higher Plants*, eds Touraev A. Forster B. Jain M. (Dordrecht: Springer), 241–263. 10.1007/978-1-4020-8854-4_21

[B24] Germanà M. A. (2011a). Anther culture for haploid and doubled haploid production. Special issue: “in vitro ploidy manipulation in the genomics era.” *Plant Cell Tiss. Org.* 104 283–300.

[B25] Germanà M. A. (2011b). Gametic embryogenesis and haploid technology as valuable support to plant breeding. *Plant Cell Rep.* 30 839–857. 10.1007/s00299-011-1061-7 21431908

[B26] Germanà M. A. Aleza P. Carrera E. Chen C. Chiancone B. Costantino G. (2013). Cytological and molecular characterization of three gametoclones of *Citrus clementina*. *BMC Plant Biol*. 13:129 10.1186/1471-2229-13-129 PMC3847870 24020638

[B27] Germanà M. A. Chiancone B. (2003). Improvement of the anther culture protocol in *Citrus clementina* Hort. ex Tan. microspore-derived embryoid induction and regeneration. *Plant Cell Rep.* 22 181–187. 10.1007/s00299-003-0669-7 12879259

[B28] Germanà M. A. Chiancone B. Lain O. Testolin R. (2005). Anther culture in *Citrus clementina*: a way to regenerate tri-haploids. *Aus. J. Agric. Res.* 56 839–845. 10.1071/AR05025

[B29] Germanà M. A. Chiancone B. Levy-Guarda N. Testillano P. S. Risueño M. C. (2006). Development of multicellular pollen of *Eriobotrya japonica* Lindl. through anther culture. *Plant Sci.* 171 718–725. 10.1016/j.plantsci.2006.07.005

[B30] Germanà M. A. Chiancone B. Padoan D. Bárány I. Risueño M. C. Testillano P. (2011). First stages of microspore reprogramming to embryogenesis through anther culture in *Prunus armeniaca* L. *Environ. Exp. Bot.* 71 152–157. 10.1016/j.envexpbot.2010.11.011

[B31] Germanà M. A. Reforgiato Recupero G. (1997). Haploid embryos regeneration from anther culture of ‘Mapo’ tangelo (*Citrus deliciosa* x *C. paradisi*). *Adv. Hort. Sci.* 11 147–152.

[B32] Germanà M. A. Scarano M. T. Crescimanno F. G. (1996). First results on isolated microspore culture of *Citrus*. *Proc. Int. Soc. Citriculture* 2 882–885.

[B33] Germanà M. A. Wang Y. Y. Barbagallo M. G. Iannolino G. Crescimanno F. G. (1994). Recovery of haploid and diploid plantlets from anther culture of *Citrus clementina* Hort. ex Tan. and *Citrus reticulata* Blanco. *J. Hort. Sci.* 69 473–480.

[B34] Höfer M. (2004). In vitro androgenesis in apple-improvement of the induction phase. *Plant Cell Rep*. 22 365–370. 10.1007/s00299-003-0701-y 14685764

[B35] Höfer M. Touraev A. Heberle-Bors E. (1999). Induction of embryogenesis from isolated apple microspores. *Plant Cell Rep.* 18 1012–1017. 10.1007/s002990050700

[B36] Hormaza H. Herrero M. (1996). Male gametophytic selection as a plant-breeding tool. *Sci. Hort.* 65 321–333. 10.1016/0304-4238(96)00899-0

[B37] Hu T. C. Kasha K. J. (1999). A cytological study of pretreatments used to improve isolated microspore cultures of wheat (*Triticum aestivum* L.) cv. *Chris. Genome* 42 432–441. 10.1139/gen-42-3-432

[B38] Kaur-Sawhney R. Flores H. E. Galston A. W. (1980). Polyamine-induced DNA synthesis and mitosis in oat leaf protoplasts. *Plant Physiol.* 65 368–371. 10.1104/pp.65.2.368 16661192 PMC440329

[B39] Kumlehn J. Serazetdinova L. Hensel G. Becker D. E. Lörz H. (2006). Genetic transformation of barley (*Hordeum vulgare* L.) via infection of androgenetic pollen cultures with *Agrobacterium tumefaciens*. *Plant Biotech. J.* 4 251–261. 10.1111/j.1467-7652.2005.00178.x 17177801

[B40] Murashige T. Skoog F. A. (1962). Revised medium for rapid growth and bioassays with tobacco tissue cultures. *Physiol. Plant.* 15 473–497. 10.1111/j.1399-3054.1962.tb08052.x

[B41] Niedz R. P. Evens T. J. (2011). The effects of benzyladenine and meta-topolin on in vitro shoot regeneration of sweet orange. *ARPN J. Agric. Biol. Sci.* 6 64–73.

[B42] Nitsch C. (1974). La culture de pollen isolé sur mileu synthètique. *C R Acad. Sci. Paris* 278 1031–1034.

[B43] Nitsch C. (1977). “Culture of isolated microspores,” in *Applied and Fundamental Aspects of Plants, Cell, Tissue and Organ Culture,* eds Reinert J. Bajaj Y. P. S. (Berlin: Springer), 268–278.

[B44] Nitsch J. P. Nitsch C. (1969). Haploid plants from pollen grains. *Science* 163 85–85. 10.1126/science.163.3862.85 17780179

[B45] Novelli V. M. Cristofani M. Souza A. A. Machado M. A. (2006). Development and characterization of polymorphic microsatellite markers for the sweet orange (*Citrus sinensis* L. Osbeck). *Genet. Mol. Biol.* 29 90–96. 10.1590/S1415-47572006000100018

[B46] Padoan D. Khan P. S. S. V. Chiancone B. Barany I. Risueno M. C. Testillano P. (2011). First stages of microspore reprogramming to embryogenesis through isolated microspore culture in eriobotrya japonica lindl. *Acta Hortic.* 887 285–289.

[B47] Prem D. Solís M. T. Bárány I. Rodríguez-Sanz H. Risueño M. C. Testillano P. S. (2012). A new microspore embryogenesis system under low temperature which mimics zygotic embryogenesis initials, expresses auxin and efficiently regenerates doubled-haploid plants in *Brassica napus*. *BMC Plant Biol.* 12:127 10.1186/1471-2229-12-127 PMC3464609 22857779

[B48] Ramírez C. Chiancone B. Testillano P. S. García-Fojeda B. Germanà M. A. Risueño M. C. (2003). First embryogenic stages of *Citrus* microspore-derived embryos. *Acta Biol. Cracov. Bot.* 45 53–58.

[B49] Ramírez C. Testillano P. S. Pintos B. Moreno M. A. Bueno M. A. Risueño M. C. (2004). Changes in pectins and MAPKs related to cell development during early microspore embryogenesis in *Quercus suber* L. *Eur. J. Cell Biol.* 83 213–225. 10.1078/0171-9335-00368 15346811

[B50] Ravikumar R. L. Patil B. S. Soregaon C. D. Hegde S. G. (2007). Genetic evidence for gametophytic selection of wilt resistant alleles in chickpea. *Theor. Appl. Genet.* 114 619–625. 10.1007/s00122-006-0462-4 17143648

[B51] Reinert J. Bajaj Y. P. S. (1977). “Anther culture: haploid production and its significance,” in *Applied and Fundamental Aspects of Plants, Cell, Tissue and Organ Culture*, eds Reinert J. Bajaj Y. P. S. (Berlin: Springer), 251–267. 10.1007/978-3-662-02279-5

[B52] Rodríguez-Sanz H. Manzanera J. A. Solís M. T. Gómez-Garay A. Pintos B. Risueño M. C. (2014). Early markers are present in both embryogenesis pathways from microspores and immature zygotic embryos in cork oak, *Quercus suber* L. *BMC Plant Biol.* 14:224 10.1186/s12870-014-0224-4 PMC4147960 25162300

[B53] Seguì-Simarro J. M. (2010). Androgenesis revisited. *Bot. Rev*. 76 377–404. 10.1007/s12229-010-9056-6

[B54] Smykal P. (2000). Pollen embryogenesis-the stress mediated switch from gametophytic to sporophytic development. *Current status* and future prospects. *Biol. Plant.* 43 481–489. 10.1023/A:1002835330799

[B55] Soleimani A. Talaie A. R. Naghavi M. R. Zamani Z. (2010). Male gametophytic and sporophytic screening of olive cultivars for salt stress tolerance. *J. Agr. Sci. Tech.* 12 173–180.

[B56] Solís M. T. Pintos B. Prado M. J. Bueno M. A. Raska I. Risueño M. C. (2008). Early markers of in vitro microspore reprogramming to embryogenesis in olive (*Olea europaea* L.). *Plant Sci.* 174 597–605. 10.1016/j.plantsci.2008.03.014

[B57] Torrigiani P. Altamura M. M. Capitani F. Serafini-Fracassini D. Bagni N. (1989). De novo root formation in thin cell layers of tobacco: changes in free and bound polyamines. *Physiol. Plant.* 77 294–301. 10.1111/j.1399-3054.1989.tb05644.x

[B58] Wang M. Van Bergen S. Van Duijn B. (2000). Insights into a key developmental switch and its importance for efficient plant breeding. *Plant Physiol.* 124 523–530. 10.1104/pp.124.2.523 11027703 PMC1539284

